# Too Much of a Good Thing? An Evolutionary Theory to Explain the Role of Ceramides in NAFLD

**DOI:** 10.3389/fendo.2020.00505

**Published:** 2020-07-31

**Authors:** Annelise M. Poss, Scott A. Summers

**Affiliations:** Department of Nutrition and Integrative Physiology, University of Utah, Salt Lake City, UT, United States

**Keywords:** fatty liver, ceramide, NASH, NAFL, NAFLD (non alcoholic fatty liver disease)

## Abstract

Non-alcoholic fatty liver disease (NAFLD), which ranges from the relatively benign and reversible fatty liver (NAFL) to the more advanced and deadly steatohepatitis (NASH), affects a remarkably high percentage of adults in the population. Depending upon severity, NAFLD can increase one's risk for diabetes, cardiovascular disease, and hepatocellular carcinoma. Though the dominant histological feature of all forms of the disease is the accumulation of liver triglycerides, these molecules are likely not pathogenic, but rather serve to protect the liver from the damaging consequences of overnutrition. We propose herein that the less abundant ceramides, through evolutionarily-conserved actions intended to help organisms adapt to nutrient excess, drive the cellular events that define NAFL/NASH. In early stages of the disease process, they promote lipid uptake and storage, whilst inhibiting utilization of glucose. In later stages, they stimulate hepatocyte apoptosis and fibrosis. In rodents, blocking ceramide synthesis ameliorates all stages of NAFLD. In humans, serum and liver ceramides correlate with the severity of NAFLD and its comorbidities diabetes and heart disease. These studies identify key roles for ceramides in these hepatic manifestations of the metabolic syndrome.

## Introduction

Non-alcoholic fatty liver disease (NAFLD) is reaching epidemic proportions, affecting 25% of the adult population worldwide (1, 2). It consists of two disease states of varying severity: the relatively benign simple fatty liver termed non-alcoholic fatty liver (NAFL) and the more severe non-alcoholic steatohepatitis (NASH). NASH can lead to hepatic scarring (cirrhosis) and fibrosis, which contribute to hepatocellular carcinoma (HCC), the most common liver cancer and the third leading cause of cancer-related death in the USA. Moreover, NAFLD increases one's risk for cardiometabolic disorders including type-2 diabetes and coronary artery disease. These interrelated cardiometabolic disorders account for a sizable proportion of global deaths and overall health expenditures.

The defining feature of NAFL is the accumulation of large lipid droplets in hepatocytes. NASH is a worsened condition characterized by additional hepatocellular ballooning, lobular inflammation, and fibrosis. These histological characteristics of NASH reveal serious tissue damage that can result in organ failure. Though the most striking histological feature of NAFLD across all stages is the accumulation of triglycerides, these lipids are likely inert markers of the condition, rather than being bioactive drivers of the pathology. Instead, studies have identified roles for sphingolipids such as ceramides in each stage of NAFLD and in many features of NASH: fat accumulation, insulin resistance, mitochondrial dysfunction, apoptosis, and fibrosis. The purpose of this review is to discuss the relevant evidence in both human and animal studies for the role of ceramides in each element of these liver pathologies. Moreover, we propose a unifying, evolutionary theory to explain why ceramides initiate these deleterious actions.

## Sphingolipid Biosynthesis and Degradation

Sphingolipids are a richly diverse lipid class, comprising over 4,000 distinct species that serve a wide variety of biological roles. Their *de novo* synthesis starts in the endoplasmic reticulum with the condensation of fatty and amino acids to produce the basic sphingoid scaffold. A series of enzymatic reactions follow that produce ceramides, which are the precursors for the more abundant sphingolipids (e.g., sphingomyelins, gangliosides). Sphingolipids play integral roles in membrane structure and fluidity as well as cellular growth and function, including initiation of a coordinated stress response and ultimately apoptosis (3). Circulating factors associated with metabolic disorders including saturated fats, inflammatory cytokines, and glucocorticoids invariably stimulate biosynthesis of ceramides (4, 5).

The first enzyme in the biosynthetic pathway is serine palmitoyl transferase (SPT), which condenses amino (e.g., serine) and fatty acids (e.g., palmitoyl-CoA) to produce the sphingoid backbone (6). The third step in the pathway, catalyzed by one of six different (dihydro)ceramide synthases (CERS1-6), accounts for much of the diversity in sphingolipids by adding acyl chains to the sphingoid scaffold (7). These enzymes show variable substrate specificities and tissue distributions. CERS2 is the primary isoform in the liver, adding the C24 and C24:1 acyl-chains. These very-long-chain ceramides appear benign. CERS5 and 6 add the C16-acyl chains, producing the ceramides that are most strongly implicated in cardiometabolic diseases. CERS6 produces C16-ceramides that contribute to NAFLD and adipose tissue dysfunction (8, 9). CERS5 appears to have a deleterious role in the heart (10). Other articles in this review series will offer a more in-depth analysis of the unique roles and distributions of distinct ceramide species.

The product of the CERS reactions, during *de novo* biosynthesis, are the dihydroceramides. Dihydroceramide desaturases introduce a double bond into the d4 position of the sphingoid backbone of dihydroceramides to produce the more abundant ceramides (11). Despite the structural similarities between ceramides and dihydroceramides, differing by only a double bond, the two molecules exhibit radically different functional roles in cellular signaling and metabolism (12, 13). Mammals contain two desaturases: a ubiquitously expressed DES1 and a skin- and gut-specific DES2 that inserts an additional hydroxyl group in the d4 position to generate phytoceramides.

Following their synthesis in the ER, ceramides and dihydroceramides traffic to the Golgi apparatus where they are converted into complex sphingolipids through the addition of various head groups (e.g., phosphocholine to produce sphingomyelin; sugar moieties to produce the glucosylceramides and gangliosides; phosphate to produce ceramide-1-phosphate; or acyl-CoAs to produce the 1-O-acylceramides). Ceramides can be re-formed by the hydrolysis of the choline head group from sphingomyelins or through a salvage pathway that involves the re-acylation of sphingosine (via CERS enzymes) produced by ceramidase-mediated ceramide degradation (14, 15). Receptors for adiponectin, an anti-diabetic and cardioprotective adipokine, are ligand-activated ceramidases (16, 17). Studies in rodents have demonstrated hepatoprotective effects of adiponectin in multiple modes of liver injury. Moreover, adiponectin is an independent risk factor for NAFLD in diverse clinical cohorts (18–22). The rate of adiponectin-stimulated ceramide degradation thus appears to influence the progression of NAFLD and other cardiometabolic diseases.

Experimental manipulations of the enzymes that control ceramide synthesis or metabolism have produced a wealth of data about the role of ceramides in in the progressive stages of NAFL and NASH, as well as associated comorbidities including insulin resistance and cardiovascular disease. These will be discussed in depth below.

## Ceramides in NAFL

NAFL, or simple fatty liver, is characterized in humans by ≥5% macrovesicular fat accumulation in hepatocytes without substantial inflammation or hepatocellular damage (1). NAFL is considered relatively benign and is asymptomatic. Studies demonstrate no direct relationship between the extent of steatosis and survival in humans (23). In the current progressive model of NAFLD development, however, NAFL precedes the more severe liver disease states such as NASH, cirrhosis and HCC.

Though increased hepatic triglyceride deposition precedes NASH, triglycerides are likely inert storage molecules that do not directly elicit the cellular dysfunction that causes NASH (24, 25). In accordance with this theory, inhibiting triglyceride synthesis at the level of DGAT2 reduces steatosis, but enhances fibrosis, oxidative stress, and lipid peroxidation (26, 27). The bioactive ceramides are more likely to drive the pathology. In accordance with this theory, a number of studies have identified correlations between ceramides and different measures of NAFLD/NASH in humans (28–37).

Interventional studies in rodents invariably demonstrate that ceramides are necessary for NAFL development. One such study was performed using mice that allow for conditional depletion of the *Degs1* gene that encodes DES1. Deletion of the gene allows the investigator to acutely replace the ceramides in tissues with dihydroceramides lacking the key double bond (13). Deleting *Degs1* from adult mice either prevented or reversed hepatic steatosis in *ob/ob* mice by blunting lipid uptake (via the fatty acid translocase CD36) and decreasing expression of lipogenic genes (e.g., *Srebf1*). Similarly, liver-specific ablation of the *Degs1* gene encoding DES1 (or liver-targeted knockdown of *Degs1* using shRNA) reversed high fat diet-induced hepatic steatosis (13). Similar findings were reported using mice that allow for inducible, liver-specific overexpression of acid ceramidase (38) or liver-specific deletion of CERS6 (9), which lower ceramides, normalize lipid uptake and metabolism, and resolve diet-induced hepatic steatosis. Thus, depleting ceramides from the liver, by either blocking their synthesis or stimulating their degradation, resolves NAFL. In each of these studies, the interventions also resolved other features of the metabolic syndrome including insulin resistance and serum hypertriglyceridemia.

Though the studies in the preceding paragraph have demonstrated that ceramides are necessary for NAFL, relatively few have evaluated whether they are sufficient to drive the pathology using gain-of-function approaches. *In vivo*, the closest has been studies by the Gonzalez laboratory, which found that intestinal Farnesoid X receptors induced ceramides which traveled to the liver to stimulate steatosis. In these studies, they administered C16-ceramides to antibiotic-treated mice, finding that they induced steatosis and increased expression of the lipogenic gene *Srebp1c* and other transcriptional markers of steatosis (e.g., *Cidea, Fasn*, etc.) (39). *In vitro*, overexpression of CERS6 overexpression in primary hepatocytes was shown to be sufficient to increase levels of C16-ceramides, induce triglyceride accumulation, impair mitochondrial respiration, and antagonize insulin signaling (40).

## Ceramides, Lipid Uptake, and Triglyceride Synthesis

**Figure 1:**
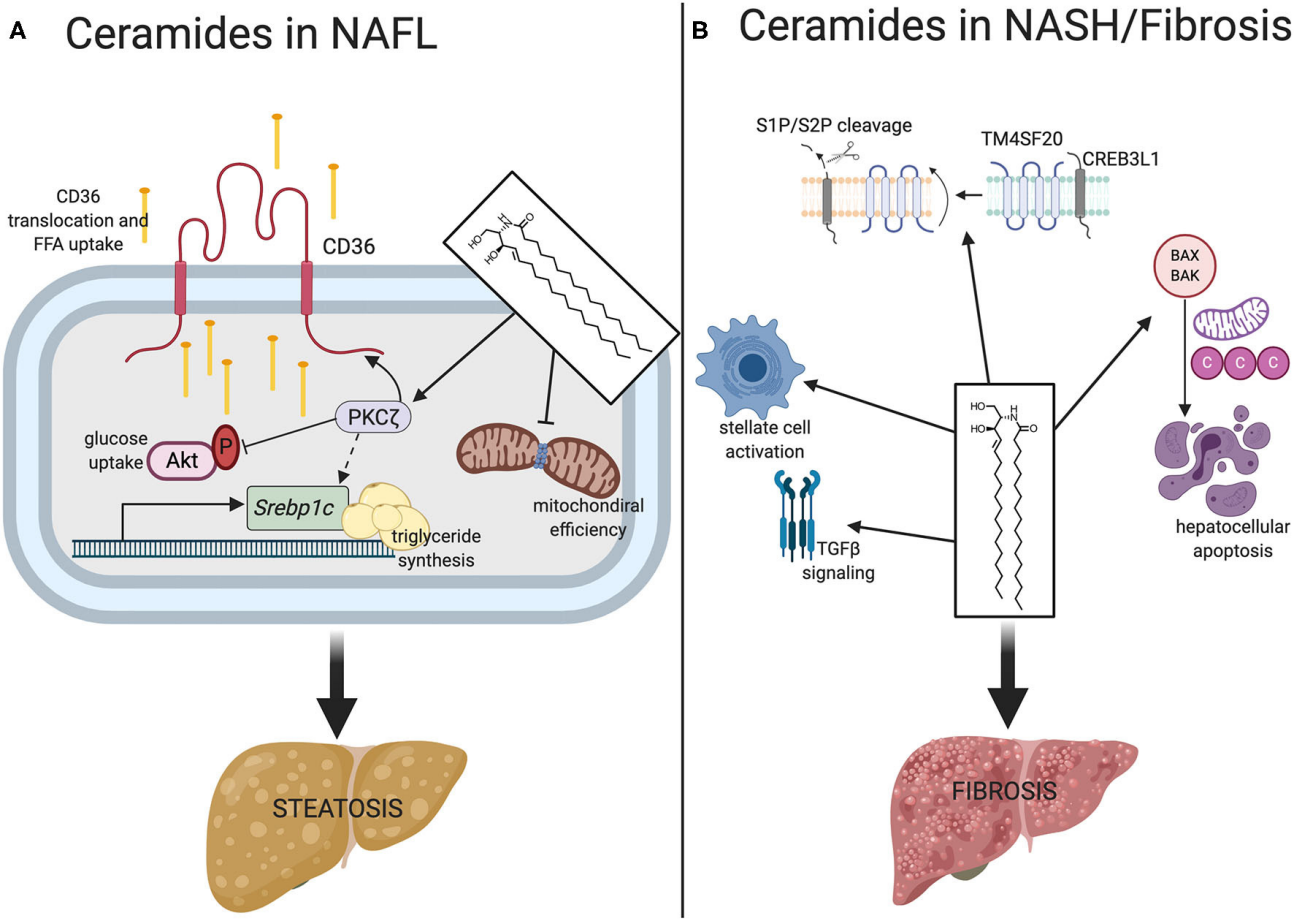
Mechanisms of ceramides contribute to NALF and NASH/fibrosis. **(A)** In NAFL, ceramides promote lipid accumulation ultimately resulting in hepatosteatosis. In promoting steatosis, ceramides stimulate the translocation of CD36 to its active site on the plasma membrane via obligate intermediate PKCζ where it facilitates uptake and esterification of free fatty acids. Ceramide action on PKCζ also upregulates transcription of *Srebpc1* and inhibits Akt. Ceramides also reduce mitochondrial efficiency, promoting fat as a preferred substrate. **(B)** Ceramides promote fibrosis through TGF-β signaling, CREB3L1 proteolysis, stellate cell activation, and hepatocellular apoptosis. *Srebpc1*, sterol regulatory element binding protein 1; S1P/S2P, site 1 protease, site 2 protease; TM4SF20, transmembrane 4 super family 20; CREB3L1, CAMP responsive element binding protein 3. Figure was created with BioRender.com.

Several mechanisms explain the ceramide-induction of hepatic steatosis (Figure 1A).

First, ceramides increase lipid uptake into the liver, at least in part by stimulating the translocation of the CD36 fatty acid translocase to the plasma membrane (13, 38). This event involves the atypical protein kinase C zeta (PKCζ) (38), a ceramide effector that has several roles in lipid and glucose metabolism. Addition of exogenous short-chain ceramide analogs activates PKCζ in H4IIe hepatocytes and overexpression of dominant negative PKCζ negates ceramide-induced CD36 translocation. In some tissues, this ceramide-PKCζ axis also impairs glucose utilization through diminished activation of Akt (protein kinase B) (41).

Second, ceramides activate signaling pathways that induce triglyceride synthesis, in part by inducing the master transcriptional regulator sterol response element binding protein (SREBP). These are potently induced by ceramides and during NAFLD pathogenesis (39). In fact, gene variants and polymorphisms of *Srebpf1* are associated with increased risk of NAFLD development (42). *In vitro*, ceramides are sufficient to stimulate hepatic *Srebpf1* gene expression, activating a number of downstream targets that facilitate triglyceride production and fatty acid elongation (43). This phenomenon is independent of intracellular cholesterol levels, mediated by ceramides regulating posttranscriptional physiological processing of *Srebpf1*. Similarly, liver specific DES1 depletion in mice displays a striking downregulation of SREBPs (13). This mechanism is likely driven through the aforementioned ceramide-PKCζ axis, which is established as a contributor to hypertriglyceridemia and hepatic steatosis in mice (44). Indeed, several studies have shown that PKCζ regulates SREBP-mediated triglyceride synthesis.

## NAFL to NASH Transition/NASH

The transition from NAFL to NASH worsens an individual's prognosis, as NASH is considerably more likely to progress to HCC and cirrhosis, and ultimately mortality, than the relatively benign NAFL (1, 45). Meta-analyses have also identified fibrosis grade as an independent risk factor for liver and cardiovascular-related mortality (46). Understanding this transition is essential for developing clinical interventions to prevent end stage liver disease and improve patient outcomes. NASH differs from NAFL in that steatosis is accompanied by lobular inflammation, hepatocellular ballooning degeneration, and fibrosis. NAFLD has a complicated bidirectional relationship with insulin resistance, which is considered a driving factor for progression to more severe liver disease states (47).

NASH patients have elevated liver ceramides and increased hepatic expression of ceramide synthesizing genes (29, 30, 32, 48, 49). Ceramides have also been observed as a signature of the NASH disease state in disparate mouse models of the condition, including those that are not obese. For example, an unbiased comparison of two diet-mediated mouse NASH models, the methionine choline deficient and the atherogenic diets, identified alterations in sphingolipid metabolism as a common feature in both (34). This study included a thorough exploration of histological, transcriptional, and lipidomic changes in both models. Despite considerable differences in the mechanisms of these two NASH models, both exhibited decreases in cholesterogenesis, transcriptional upregulation of ceramide synthases, and increased *de novo* sphingolipid synthesis resulting in elevated ceramides, dihydroceramides, and glucosylceramides. In accordance, NASH patients that undergo a weight loss-based lifestyle intervention that reverses the condition show a reduction in circulating ceramides and hepatic expression of pro-ceramide synthesizing genes (32).

**Figure 2:**
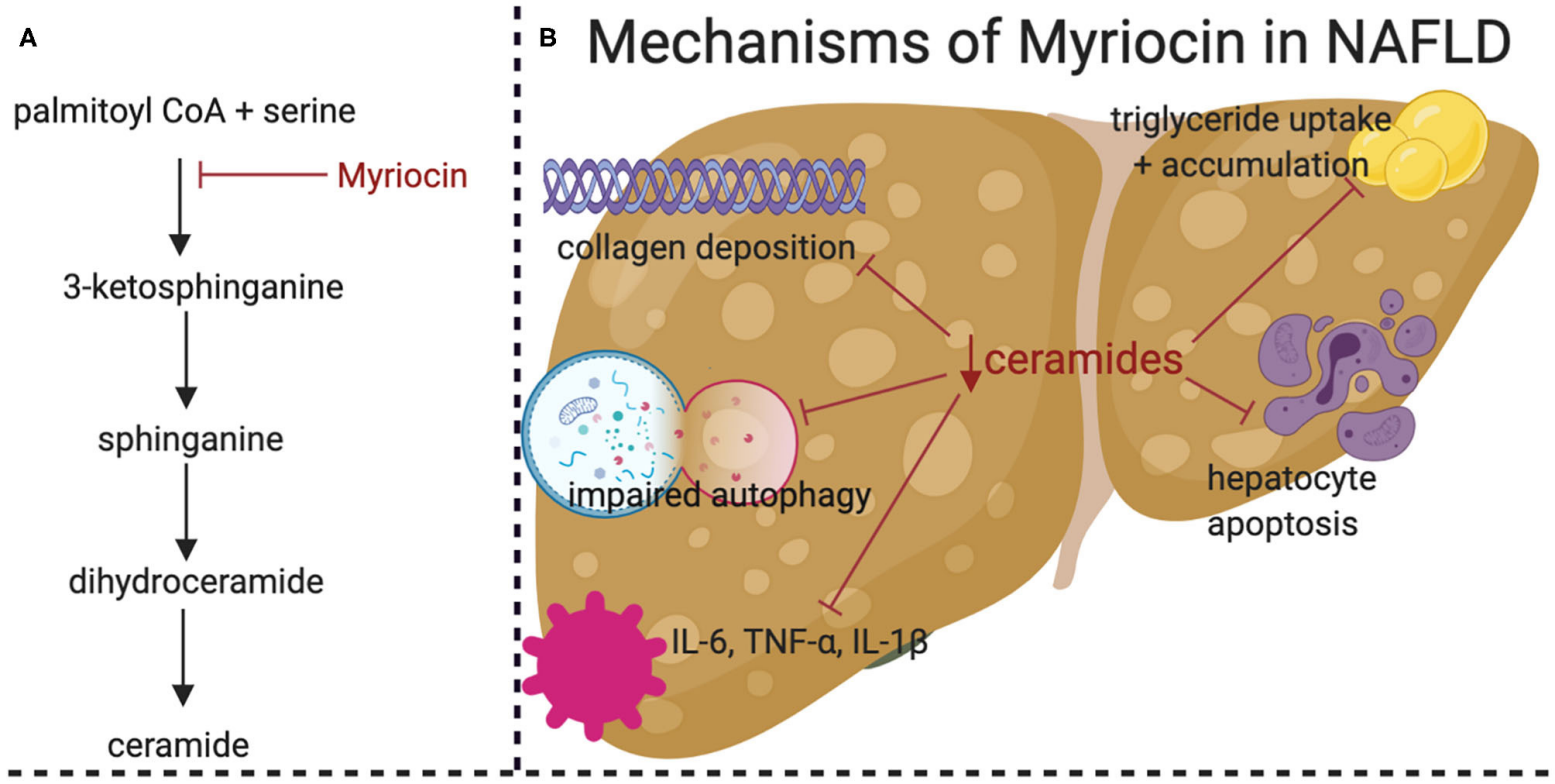
Pharmacological inhibition of *de novo* ceramide synthesis prevents high fat diet (HFD) induced NASH. **(A)** Simplified schematic of ceramide *de novo* synthesis pathway, demonstrating the target of Myriocin (in red). **(B)** Graphical summary of the beneficial effects of Myriocin treatment in HFD-fed Sprague-Dawley rats. HFD-induced NAFLD related effects are shown in black with the beneficial effects of Myriocin treatments shown with red lines. Figure was created with BioRender.com.

In rodents, several studies have shown that pharmacological reduction of ceramides prevents NASH onset (Figure 2). The majority have utilized myriocin, which is a potent and irreversible inhibitor of serine palmitoyltransferase. One such study used high fat diet feeding in adult Sprague-Dawley rats and demonstrated that myriocin prevented steatosis and fibrosis (50). In this experiment, ceramide depletion led to inactivation of signaling by c-Jun N-terminal kinase (JNK), a kinase implicated in inflammatory responses and the regulation of apoptosis. Moreover, myriocin normalized levels of inflammatory cytokines such as TNF-α, IL-1β, and IL-6. A similar study, also conducted in Sprague-Dawley rats fed a high fat diet, demonstrated that myriocin normalized body weight, serum transaminase, and serum triglycerides (28). Studies in cultured hepatocytes showed that myriocin normalized expression profiles of genes involved in fatty acid metabolism and restored autophagy. A final study performed in Wistar rats demonstrated that myriocin treatment for 7 days following establishment of high fat diet induce NAFLD was sufficient to lower ceramides, improve whole body insulin tolerance, and reduce hepatic steatosis (51). Ceramides are also elevated in livers of mice exposed to carbon tetrachloride, a toxin that is commonly used to mimic NASH, fibrosis, and cirrhosis (52).

Unfortunately, no studies involving direct manipulation of ceramide synthesizing genes to increase or decrease ceramides in the context of NASH have been published to date.

## Ceramides, Cell Death, and Fibrosis

Apoptosis is an essential element in the cascade of events that cause NASH. In both *in vitro* and *in vivo* models, administration of pan-caspase inhibitors or targeted genetic ablation of specific caspases suppresses apoptosis, and fibrosis (53). Ceramides have long-been known to induce apoptosis, as they increase mitochondrial outer membrane permeability to cytochrome C, leading to caspase activation (54). Blocking ceramide production negates the pro-apoptotic effects of many different cellular insults (55), including the saturated fatty acids and cytokines implicated in NASH. Ceramides have also been demonstrated to induce hepatocellular necrosis through induction of mitochondrial failure (56).

Ceramides alter mitochondrial membrane permeability by several different mechanisms. They recruit the pro-apoptotic protein BAX to mitochondria, where it oligomerizes and increases membrane permeability (57). Ceramides bind voltage-dependent anion channel 2, which further increases mitochondrial outer membrane permeability (58). Ceramides, through extensive hydrogen bonding with themselves, are capable of forming channels in membranes that allow passage of small proteins (59–62). And, ceramides inhibit pro-survival signaling by the protein kinase Akt/PKB (63). This latter effect on Akt/PKB accounts for the ability of ceramides to induce insulin resistance (63, 64).

Ceramides may also directly participate in the fibrosis signaling pathways that control collagen deposition (Figure 1B). In fact, hepatic stellate cells, the major extracellular matrix producing cells of the liver, exhibit increased ceramide concentrations and ceramide synthesizing gene transcripts upon activation in culture (65). This suggests that bioactive lipids such as ceramides may affect hepatic stellate cell activation and hepatic collagen deposition during NASH. Though the precise mechanisms remain elusive, ceramides are positive regulators of signaling by TGFβ, a major pro-fibrogenic cytokine (66–68). In addition, ceramides enhance cleavage of plasma membrane resident CREB3L1 by site-1 or site-2 proteases, a process that liberates a protein fragment that enters the nucleus to bind Smad4 and activate transcription of genes required for assembly of collagen-containing extracellular matrix (69, 70). This process, termed regulated intramembrane proteolysis, results from ceramides altering the orientation of TM4SF20, a protein that, in the absence of ceramides, blocks access of the proteases to CREB3L1 (70, 71).

## Ceramides in NAFLD Associated Comorbidities

NALFD is the hepatic manifestation of the metabolic syndrome. Ranging from a simple fatty liver to the far more severe NASH, the conditions increase risk for diabetes, cardiovascular disease, hepatocellular carcinoma and liver failure. In fact, cardiovascular disease is the most common cause of death in NAFLD patients, accounting for more than twice the number of deaths than progressive liver related complications (1, 72). Even in the absence of metabolic syndrome, NAFLD patients experience increased risk of cardiovascular disease (73).

Studies with knockout mice reveal that the liver provides >50% of serum ceramides (13). Interestingly, measurement of ceramides and related sphingolipids in serum has proven to have clinical utility, serving as potent, cholesterol-independent prognostic biomarkers of cardiovascular disease incidence and mortality (74–79). Circulating ceramides have also been associated with insulin resistance and type 2 diabetes in large clinical cohorts (80–82). Though the literature is largely devoid of large studies relating circulating ceramides to NAFLD, the noted relationships between serum and hepatic ceramides as with established NAFLD and as a distinction between NAFL and NASH strongly suggests that their measurement could have utility for diagnosing the condition (30, 32, 33).

A large proportion of patients with NAFL express a common gene variant (i.e., rs738409) in Patatin-like phospholipase domain-containing protein 3 (PNPLA3). The mutation in this triglyceride lipase gene leads to marked changes in liver fat. Unlike individuals with the “metabolic NAFLD” described in this review, those with PNPLA3-induced NAFLD often remain metabolically healthy, with less insulin resistance, diabetes, and cardiovascular disease (83, 84). Interestingly, ceramide concentrations are not elevated in individuals with “PNPLA3 NAFLD.” The authors attributed the lack of hepatic ceramide in subjects with the PNPLA3 variant to their ability to effectively store fat as triglycerides, rather than letting it be diverted into the sphingolipid pathway. Notably, PNPLA3 mutant patients can still develop severe fibrosis (85), suggesting ceramide-independent modes of fibrogenesis exist, in addition to ceramide-dependent ones.

Collectively, these studies indicate that ceramides are markers of NAFLD that play causative roles in several disease features. While more work is needed to determine the precise role of ceramides in NASH pathogenesis, the data thus far strongly support development of ceramide-lowering strategies to combat these liver pathologies.

## An Evolutionary Basis for the Role of Ceramides in NAFLD

We've discussed a number of ceramide-driven mechanisms that influence the health of the hepatocyte under conditions of nutrient overload. We speculate that ceramides serve as nutritional signals, accumulating when the triglyceride stores are saturated and the cellular energy needs are met. The cells respond to the increasing ceramides by initiating actions which protect them from detergent-like FFAs (86). Indeed, each of the ceramide mechanisms we have identified protect cellular membranes from the destabilizing actions of these lytic fatty acids.

In the early stages of disease progressions (e.g., NAFL), ceramides initiate actions that promote the safe uptake of fatty acids through the cellular membrane and facilitate their storage as triglycerides. For example, they increase the allotment of fatty acid translocases (e.g., CD36 or fatty acid binding proteins) on the cellular membrane, which facilitates safe passage of FFAs through the bilayer and speeds their esterification. In parallel, ceramides induce SREBP and its target genes (e.g., DGATs) to enable the incorporation of fatty acids into triglycerides. Ceramides also reduce glucose utilization by inhibiting Akt/PKB, thus allowing cells to switch to fatty acids as a preferred energy source. Lastly, they decrease mitochondrial efficiency, thus maximizing the number of fatty acids that can be oxidized while minimizing the effect on mitochondrial membrane potential. All of these actions, which were at one time likely intended to protect the cell from fatty acid toxicity, underlie NAFL.

As ceramide levels continue to rise, they initiate actions to protect the organism from the compromised, lipid-laden cells. For example, we hypothesize that ceramide-mediated apoptosis and fibrosis are extensions of the aforementioned signaling mechanisms that protect the organism when cells are compromised. For example, ceramide induction of apoptosis allows for a controlled cell death, preventing the release of cytosolic content into the extracellular space. Similarly, the induction of fibrosis allows the organism to protect itself from a damaged region of tissue. Thus, ceramides protect the organism from damage resulting from uncontrolled membrane lysis.

This theory—that ceramides signal cellular events to combat the burden of toxic levels of free fatty acids—provides a unifying framework and testable hypotheses about their role in disease.

## Conclusions

The published data thus far clearly establish roles for ceramides as drivers of different features NAFLD. Nonetheless, additional work is needed. First, more comprehensive assessments of the roles of ceramides in the terminal steps of NASH (e.g., apoptosis and fibrosis) in rodent models are warranted. These should include investigations of whether ceramide-lowering is sufficient to reverse the pathology at the later stages of disease progression. Moreover, it should include genetic manipulations to discern which effects are due to autonomous actions within the hepatocyte. Second, additional clinical verifications, including lipidomics of large liver biobanks and genomic determinations of genes that influence ceramides, would support efforts to develop this therapeutic approach. Despite these areas of need, the evidence is strong that ceramides contribute to NAFLD. Therapeutic approaches that selectively lower ceramides could show enormous progress as a means of combating NAFLD. Ceramides merit a sustained and rigorous evaluation from the scientific community.

## Author Contributions

AP and SS drafted the manuscript in collaboration with one another. AP generated the figures. All authors contributed to the article and approved the submitted version.

## Conflict of Interest

SS is a consultant and shareholder with Centaurus Therapeutics. The remaining author declares that the research was conducted in the absence of any commercial or financial relationships that could be construed as a potential conflict of interest.
